# Redox Homeostasis and Antioxidant Strategies in the Pathophysiology

**DOI:** 10.3390/antiox13030281

**Published:** 2024-02-26

**Authors:** Alessia Remigante, Marika Cordaro, Rossana Morabito

**Affiliations:** 1 Department of Chemical, Biological, Pharmaceutical and Environmental Sciences, University of Messina, 98166 Messina, Italy; rmorabito@unime.it; 2 Department of Biomedical, Dental and Imaging Sciences, University of Messina, 98166 Messina, Italy; marika.cordaro@unime.it

The molecular mechanisms underlying oxidative stress, and pathophysiological consequences in cell and tissue function, are frequently described as the imbalance between the production of reactive species and the ability to defend through sophisticated antioxidant machinery (Contributions 1–3). A certain amount of oxidative stress is useful to the body for growth and cell signaling. However, excess levels, produced by several endogenous and exogenous processes, exert deleterious effects on cell components, including proteins lipids and nucleic acids, altering the redox status of the cell (Contribution 4). Antioxidants may protect against cell damage during oxidative stress (Contribution 5). New research showed that natural antioxidants in foods commonly produce better health and life quality (Contribution 6). Dietary or natural antioxidants play an important role in helping endogenous antioxidants to scavenge excess free radicals (Contributions 7,8). Antioxidant supplements include several important substances (carotenoids, polyphenols, phycocyanin and flavonoids), which are rich in vegetables, fruits, and natural foods (Contributions 8,9).

This Special Issue has been developed to collect and contribute to the dissemination of novel findings focused on the investigation of the molecular mechanisms triggered by oxidative stress, aiming to open new avenues in therapy against oxidative damage. To this end, the beneficial effects of antioxidants and possible mechanisms of cell adaptation in the context of an imbalance of chronic oxidative-related diseases are worthy of note. Here, we offer an overview of the content of this Special Issue, which collects 1 review and 4 original articles.

Great emphasis is placed on the mitochondria which are responsible for the majority of cellular ATP production through the process of oxidative phosphorylation (OXPHOS). Cojocaru and collaborators (Contribution 10) reviewed the pathophysiological changes in and mitochondrial phenomena associated with metabolic pathologies such as diabetes, obesity, hypertension, neurodegenerative diseases, cellular aging, and cancer. Moreover, new pharmaceutical strategies that can improve the prognosis of these conditions are being investigated and developed. Several studies have highlighted the beneficial effects of using antioxidants in pathologies; vitamin E, vitamin C, coenzyme Q, and NAC administration are indicated for their oxidative stress reduction effect. However, in some clinical trials, the results of their benefits are contradictory. Due to the adverse effects of conventional antioxidants at the cellular level, the production and use of mitochondria-targeted antioxidants are becoming more relevant. The authors highlighted the genetic changes identified at the mtDNA level and selected several representative biomarkers involved in oxidative stress, summarizing the progress of therapeutic strategies. 

To study the effects of aging-related oxidative mechanisms and antioxidant strategies at the cellular level, blood cells were taken as models. Specifically, the redox balance of peripheral leukocytes determines their function and correlates with the ImmunolAge quantified by the Immunity Clock in humans. For this reason, in the study performed by Diaz-Del Cerro and co-authors (Contribution 11), the first objective was to determine whether several components of the glutathione cycle glutathione reductase (GR) and glutathione peroxidase (GPx) activities, and concentrations of oxidized glutathione (GSSG) and reduced glutathione (GSH) in leukocytes, were associated with biological age (ImmunolAge), estimated using the Immunity Clock in 190 men and women. The objective of the authors was to identify the best blood fraction (whole blood, blood cells, erythrocytes, or plasma) in order to quantify these components and correlate them with the estimated ImmunolAge. The results showed that the oxidative state of peripheral leukocytes correlated with their functionality, supporting the idea that this was the basis of immune senescence. In blood, the correlations were more significant in the fraction of blood cells with respect to ImmunolAge (positive correlations with GSSG concentration and the GSSG/GSH ratio, and negative correlations with GPx and GR activities). Therefore, blood cells are proposed as the most effective samples with which to estimate the biological age of individuals in clinical settings. 

Moreover, the loss of deformability and structural rearrangements of cytoskeletal proteins in red blood cells due to oxidative conditions represent the trigger for cellular aging processes. In particular, the function and expression of Band 3 protein (B3p) (Contribution 12), one of the most peculiar erythrocyte proteins, has become an interesting target for the study of aging. To this end, the study in question, performed by Spinelli and co-authors (Contribution 13), aimed to verify the beneficial role of Açaì extract in a D-Galactose (D-Gal)-induced model of aging in human RBCs. Specifically, B3p phosphorylation, spectrin, ankyrin, and/or protein 4.1 were analyzed in RBCs treated with 100 mM D-Gal for 24 h, with or without pre-incubation with a 10 μg/mL Açaì extract for 1 h. The authors clearly demonstrate that during natural aging the maintenance of the biconcave shape of RBCs is fundamental to counteracting exogenous stressors, including age-related oxidative stress. In addition, the early application of antioxidant molecules might be helpful to counteract oxidative stress-induced derangements. Therefore, the authors propose the use of Açaì extract against aging-related oxidative stress on a cellular level.

Oxidative stress plays a crucial role in cardiac and vascular abnormalities in different types of cardiovascular diseases. Calcific aortic stenosis (CAS) can drive vascular complications in type 2 diabetes mellitus (T2DM). In this contest, Corbacho-Alons and co-authors (Contribution 14) assessed the global oxidative status in plasma from patients with CAS, both alone and with T2DM (and when under treatment with metformin), using multi-marker scores of systemic oxidative damage (OxyScore) determined by measuring carbonyls, oxidized LDL (oxLDL), 8-hydroxy-20-deoxyguanosine (8-OHdG), and xanthine oxidase (XOD) activity, and antioxidant defense (AntioxyScore) determined through the catalase (CAT) and superoxide dismutase (SOD) activity, as well as the total antioxidant capacity (TAC). From this study, the authors highlighted the pharmacological role of metformin against oxidative stress in patients with CAS and T2DM. Thus, reducing oxidative stress or enhancing antioxidant capacity through specific therapies could be a robust strategy for managing CAS, focusing on personalized medicine. 

Elevated oxidative stress represents a striking aggravating factor of ischemic brain injury. AKR is an enzyme superfamily of NAD(P)(H)-dependent oxidoreductases, and aldo-keto reductase (AKR1C15) is present in the brain. Yang and co-authors (Contribution 15) reported for first time that the exogenous intraperitoneal administration of recombinant AKR1C15 is protective in a mouse ischemic stroke model, while abolishing ischemic preconditioning (IPC)-afforded protection. Mouse ischemic stroke and IPC were established with middle cerebral artery occlusion (MCAO) for 1 h or 12 min, respectively. The results confirmed that AKR1C15 is protective in cultured neurons and brain microvascular endothelial cells (BMVECs), and that it exerts anti-inflammatory properties in cultured microglia. Therefore, AKR1C15 could be a promising agent for the treatment of ischemic stroke.

In conclusion, the scientific publications collected highlight the multitude of experimental models and approaches currently used to investigate the potential harmful effects caused by increased oxidative stress. While acknowledging the limited scope of this collection, the guest editors hope that this editorial exposition will contribute to the field of cells responses to elevated oxidant stress. Thus, understanding oxidative stress-related disease pathophysiology could enable (1) the identification of novel therapeutic targets and (2) the proposal that these natural substances be used as potential antioxidant strategies for the treatment and prevention of disease risk.

## List of Contributions

1. Sharma, V.; Mehdi, M.M. Oxidative stress, inflammation and hormesis: The role of dietary and lifestyle modifications on aging. *Neurochem. Int.* **2023**, *164*, 105490. https://doi.org/10.1016/j.neuint.2023.105490.
2. Birben, E.; Sahiner, U.M.; Sackesen, C.; Erzurum, S.; Kalayci, O. Oxidative stress and antioxidant defense. *World Allergy Organ. J.* **2012**, *5*, 9–19. https://doi.org/10.1097/WOX.0b013e3182439613.
3. Burton, G.J.; Jauniaux, E. Oxidative stress. *Best Pract. Res. Clin. Obstet. Gynaecol.* **2011**, *25*, 287–299. https://doi.org/10.1016/j.bpobgyn.2010.10.016.
4. Sharifi-Rad, M.; Anil Kumar, N.V.; Zucca, P.; Varoni, E.M.; Dini, L.; Panzarini, E.; Rajkovic, J.; Tsouh Fokou, P.V.; Azzini, E.; Peluso, I.; et al. Lifestyle, Oxidative Stress, and Antioxidants: Back and Forth in the Pathophysiology of Chronic Diseases. *Front. Physiol.* **2020**, *11*, 694. https://doi.org/10.3389/fphys.2020.00694.
5. Jomova, K.; Raptova, R.; Alomar, S.Y.; Alwasel, S.H.; Nepovimova, E.; Kuca, K.; Valko, M. Reactive oxygen species, toxicity, oxidative stress, and antioxidants: Chronic diseases and aging. *Arch. Toxicol.* **2023**, *97*, 2499–2574.
6. Pizzino, G.; Irrera, N.; Cucinotta, M.; Pallio, G.; Mannino, F.; Arcoraci, V.; Squadrito, F.; Altavilla, D.; Bitto, A. Oxidative Stress: Harms and Benefits for Human Health. *Oxid. Med. Cell. Longev.* **2017**, *2017*, 8416763. https://doi.org/10.1155/2017/8416763.
7. Rahaman, M.M.; Hossain, R.; Herrera-Bravo, J.; Islam, M.T.; Atolani, O.; Adeyemi, O.S.; Owolodun, O.A.; Kambizi, L.; Dastan, S.D.; Calina, D.; et al. Natural antioxidants from some fruits, seeds, foods, natural products, and associated health benefits: An update. *Food Sci. Nutr.* **2023**, *11*, 1657–1670. https://doi.org/10.1002/fsn3.3217.
8. Foudah, A.I.; Salkini, M.A.; Yusufoglu, H.S.; Alkreathy, H.M.; Khan, R.A. Protective Effects of a Polyphenolic Phytochemical Quercetin against Oxidative Dysfunctions in Rats. *Evid. Based Complement. Alternat. Med.* **2023**, *2023*, 7858718. https://doi.org/10.1155/2023/7858718.
9. Lang, Y.; Gao, N.; Zang, Z.; Meng, X.; Lin, Y.; Yang, S.; Yang, Y.; Jin, Z.; Li, B. Classification and antioxidant assays of polyphenols: A review. *J. Future Foods* **2024**, *4*, 193–204.
10. Cojocaru, K.A.; Luchian, I.; Goriuc, A.; Antoci, L.M.; Ciobanu, C.G.; Popescu, R.; Vlad, C.E.; Blaj, M.; Foia, L.G. Mitochondrial Dysfunction, Oxidative Stress, and Therapeutic Strategies in Diabetes, Obesity, and Cardiovascular Disease. *Antioxidants* **2023**, *12*, 658. https://doi.org/10.3390/antiox12030658.
11. Diaz-Del Cerro, E.; Martinez de Toda, I.; Felix, J.; Baca, A.; De la Fuente, M. Components of the Glutathione Cycle as Markers of Biological Age: An Approach to Clinical Application in Aging. *Antioxidants* **2023**, *12*, 1529. https://doi.org/10.3390/antiox12081529.
12. Remigante, A.; Spinelli, S.; Pusch, M.; Sarikas, A.; Morabito, R.; Marino, A.; Dossena, S. Role of SLC4 and SLC26 solute carriers during oxidative stress. *Acta Physiol.* **2022**, *235*, e13796. https://doi.org/10.1111/apha.13796.
13. Spinelli, S.; Straface, E.; Gambardella, L.; Caruso, D.; Falliti, G.; Remigante, A.; Marino, A.; Morabito, R. Aging Injury Impairs Structural Properties and Cell Signaling in Human Red Blood Cells; Acai Berry Is a Keystone. *Antioxidants* **2023**, *12*, 848. https://doi.org/10.3390/antiox12040848.
14. Corbacho-Alonso, N.; Rodriguez-Sanchez, E.; Sastre-Oliva, T.; Mercado-Garcia, E.; Perales-Sanchez, I.; Juarez-Alia, C.; Lopez-Almodovar, L.F.; Padial, L.R.; Tejerina, T.; Mourino-Alvarez, L.; et al. Global Oxidative Status Is Linked to Calcific Aortic Stenosis: The Differences Due to Diabetes Mellitus and the Effects of Metformin. *Antioxidants* **2023**, *12*, 1024. https://doi.org/10.3390/antiox12051024.
15. Yang, T.; Li, Q.; Fadoul, G.; Alraqmany, N.; Ikonomovic, M.; Zhang, F. Aldo-Keto Reductase 1C15 Characterization and Protection in Ischemic Brain Injury. *Antioxidants* **2023**, *12*, 909. https://doi.org/10.3390/antiox12040909.

